# Fabrication of Liquid Crystalline Polyurethane/Polyhedral Oligomeric Silsesquioxane Nanofibers via Electrospinning

**DOI:** 10.3390/ma16237476

**Published:** 2023-12-01

**Authors:** Artur Bukowczan, Konstantinos N. Raftopoulos, Krzysztof Pielichowski

**Affiliations:** Department of Chemistry and Technology of Polymers, Cracow University of Technology, Warszawska 24, 31-155 Kraków, Poland; konstantinos.raftopoulos@pk.edu.pl (K.N.R.); kpielich@pk.edu.pl (K.P.)

**Keywords:** liquid crystallinity, POSS, polyurethanes, nanofibers, electrospinning

## Abstract

A series of fibrous meshes based on liquid crystalline polyurethane/POSS composites were prepared. Two types of polyhedral oligomeric silsesquioxanes (POSSs) of different structures were chosen to show their influence on electrospun fibers: aromatic-substituted Trisilanolphenyl POSS (TSP-POSS) and isobutyl-substituted Trisilanolisobutyl POSS (TSI-POSS) in amounts of 2 and 6 wt%. The process parameters were selected so that the obtained materials showed the highest possible fiber integrity. Moreover, 20 wt% solutions of LCPU/POSS composites in hexafluoroisopropanol (HFIP) were found to give the best processability. The morphology of the obtained meshes showed significant dependencies between the type and amount of silsesquioxane nanoparticles and fiber morphology, as well as thermal and mechanical properties. In total, 2 wt%. POSS was found to enhance the mechanical properties of produced mesh without disrupting the fiber morphology. Higher concentrations of silsesquioxanes significantly increased the fibers’ diameters and their inhomogeneity, resulting in a lower mechanical response. A calorimetric study confirmed the existence of liquid crystalline phase formation.

## 1. Introduction

Among all techniques used in fiber preparation, electrospinning has attracted broad interest due to its ability to form fibers with diameters down to the nanoscale. It can be applied to various types of polymers and composites by dissolving them in a suitable solvent or being applied directly from their melt [1]. Various types of nanofiber structures were described in the literature, from core–shell [2], hollow [3], and helical [4] to highly porous [5,6], which is what makes them interesting for medical [7], membrane [8], and electronic [9] applications. Moreover, liquid crystals and liquid crystalline polymers have been processed using the electrospinning technique [10], and electroactive [11,12], thermoresponsive [13], photosensitive [14,15], and core–shell fibers [16,17] are examples of the nanofibrous materials reported to be novel multi-functional materials with liquid crystalline phases.

Despite the fact that nano-structured fibers have been applied in many fields of research, there is still room for improvement in their processability or the creation of enhanced mechanical and thermal properties or new unique functions [18,19,20]. A common approach in this direction is modification via nanomoieties.

Combining crystalline properties with anisotropic orientation makes liquid crystalline polymers a unique class of materials. Over the past few decades, such polymers have become one of the most interesting matrices for the fiber industry [21,22,23]. Therefore, it is not surprising that researchers have also decided to use their potential in the field of nanotechnology, creating multifunctional nanocomposites [24]. Earlier studies have shown that the incorporation of carbon nanotubes, metal nanoparticles, or silica-based nanostructures can enhance their liquid crystalline phase transition performance, thermal stability, or compatibility [25].

POSS molecules are one of the most studied nanostructures in the field of polymer composites [26,27,28,29,30]. A few nanometers in size, the cage-like arrangements of Si-O linkages with the possibility of introducing various functional groups make POSS a widely applicable nanomaterial. Its unique structure allows the formation of covalent bonds with polymeric backbones in different configurations such as pendant, endcap, bead-like, or chain junction configurations [31]. Enhanced thermal stability, better processability, higher mechanical modulus, and improved biocompability are some of the reasons why POSS–polymer composites [32,33,34] have attracted interest from the research community. From the perspective of electrospinning, the presence of the silsesquioxane molecules can influence the process itself by enhancing conductivity and reducing the viscosity of polymeric mixtures. As a consequence, fibers with lower diameters and better orientation can be formed [35,36,37]. All the above-mentioned advantages of applying POSS molecules were already used in the electrospinning of materials intended for catalytic [38,39,40], lithium-ion battery [36,41,42], membrane [27,43,44], and biological applications [45,46,47,48,49].

Our research is focused on the preparation of liquid crystalline polyurethane (LCPU)/POSS nanofibers using composite elastomer as a raw material. The electrospinning of polyurethane/POSS composites introduced as physical blends [50] and chemical modifiers [51] is already discussed in the literature. The enhancement of mechanical and biocompatibility properties has been described. Those works create space for new research considering issues like the preparation of liquid crystalline polyurethane fibers and the analysis of the influence of POSS nanomolecules on the electrospinning process and final properties of obtained meshes. In this work, we investigate the influence of POSS molecules covalently bonded with polyurethane macrochains on the morphology and mechanical and thermal properties of fibers prepared via electrospinning.

## 2. Materials and Methods

### 2.1. Materials

**Figure 1:**
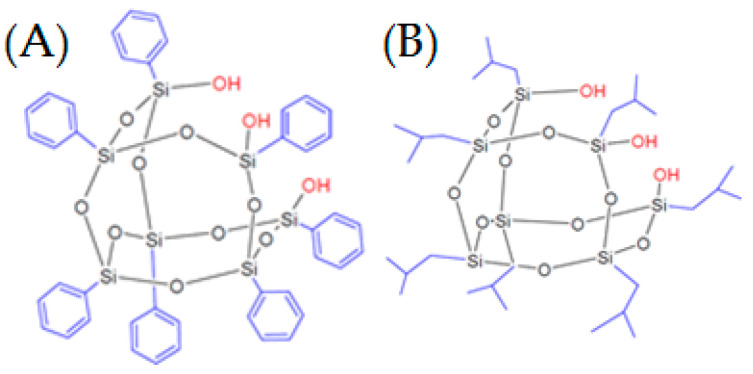
POSS structures: (**A**) TSPPOSS; (**B**) TSIPOSS.

The synthesis of liquid crystalline polyurethanes with POSS has already been described in [52,53], along with a detailed chemical characterization. In brief, a series of liquid crystalline polyurethanes containing two types of nanofiller were prepared. Using a two-stage method, firstly, a calculated amount of MDI (Methylene diphenyl diisocyanate) and PTMG (poly(tetramethylene ether)glycol) with molar mass of ~650 and POSS in amounts of 0, 2, and 6 wt% were stirred (200 rpm) at a temperature of 80 °C, and then a mesogenic unit (4,4-dihydroxyhexyloxybisphenyl) was added as a chain extender in an amount of 15 wt% based on the weights of all components. The synthesis and characterization of this mesogenic unit were reported in Ref. [52]. The reaction was conducted in an inert atmosphere. The POSSs used in this study possess three OH functionalities, which allow them to act as chemical crosslinks (Figure 1). They differ, however, in their inert vertex groups. Namely, one of them (TSPPOSS) is equipped with aromatic phenyl vertex groups, whereas the second contains aliphatic, isobutyl vertex groups.

For the preparation of nanofibers, LCPU/POSS composites were dissolved in hexafluoroisopropanol (HFIP) to obtain 10 mL of 20 wt% solutions because, in test procedures, lower concentrations caused the formation of beads. The solutions were placed in 20 mL syringes equipped with steel needles of 1 mm in diameter before being connected to a charge Master SIMCO ION voltage source. The electrospinning process was carried out at 30 °C, with an applied voltage of 10 kV and a flow rate of 3 mL/h. The obtained fibers were collected on a rotating disc collector covered with aluminum foil with a needle-to-collector distance of 10 cm. The humidity level during the process was 55%. The process was carried out for 3 h and led to the formation of fibrous meshes, which were then dried for 48 h at 50 °C under vacuum.

### 2.2. Methods

#### 2.2.1. Scanning Electron Microscopy (SEM)

The morphology of the obtained fibers was studied using a JEOL InTouchScope JSM-6010LV scanning electron microscope (JEOL Ltd., Tokyo, Japan) scanning electron microscope. In order to assess the distribution of POSS nanoparticles, an analysis of silicon distribution (mapping) was performed in selected areas of the samples using energy-dispersive X-ray spectroscopy (EDS). For the determination of the average fiber diameter, a minimum of 10 microphotographs were taken and analyzed using ImageJ software v. 1.51w [54].

#### 2.2.2. Differential Scanning Calorimetry (DSC)

In order to determine the liquid crystalline effects, a heating and a cooling run was performed in the range of −65 to 180 °C with a rate of 10 K/min. The samples were prepared in standard aluminum pans. A single layer of the electrospun mat of approximately 0.1 mm was used for the measurement in order to minimize heat transfer lags, therefore the mass was quite low, in the order of 1 mg. The analysis was performed on a Mettler Toledo 823 Differential scanning calorimeter (Mettler-Toledo, Columbus, OH, USA).

#### 2.2.3. X-ray Diffraction (XRD)

The crystalline structure was evaluated with a Bruker 2D Phaser diffractometer (Bruker, Bilerica, MA, USA). The measurement of the 2θ angles was made in the range of 5–40° using a 0.1 mm wide slit and a 1 mm shutter. The counting time was 0.5 s. A standard copper anode with a radiation wavelength of λ = 1.54184 Å was used.

#### 2.2.4. SAXS

The SAXS measurements were performed with a SmartLab SE instrument (Rigaku, Tokyo, Japan), an X-ray powder diffractometer equipped with a semiconductor (2D) X-ray detector Hypix 400 (Rigaku, Tokyo, Japan), and a 2.2 kW Cu LFF (Long Fine Focus) (Canon Electron Tubes & Devices Co., Otawara, Japan) anode lamp. The nominal 2*θ* range was −0.005° to 2° at a step of 0.002°. An empty holder measurement was taken in order to record background data, which was then subtracted from the sample intensity, taking into account the transmission of the sample:

(1) $$I\left(\theta \right)=\frac{I_{h}\left(0\right)}{I_{s}\left(0\right)}I_{s}\left(2\theta \right)-I_{h}\left(2\theta \right)$$

where “I” means intensity, and indices s and h denote the sample and holder, respectively. Normalization was performed to the thickness of the sample, and 2*θ* was converted to momentum transfer q as q=4π/λ sinθ, where λ=1.5406 Å, the wavelength of the source.

#### 2.2.5. Dynamic Mechanical Analysis (DMA)

Measurements were carried out in dynamic conditions in the temperature range of −80–80 °C at a heating rate of 2 K/min and in an inert gas atmosphere. Measurements were made for the frequencies 1; 2.5; 5; 10 Hz with target amplitude A=50 µm, maximum dynamic force $F_{d}=5\;N$, and static force $F_{f}=1.1F_{d}$. The analysis was performed using a DMA 242C NETZSCH apparatus (NETZSCH, Selb, Germany).

#### 2.2.6. Tensile Strength

Fibrous mat samples in the form of paddles were tested according to the ISO 527-2 5A [55] and ISO 37 2 standards [56]. Measurements were made for 5 samples from each series in the stretching mode at room temperature using a Brookfield CT3 texture analyzer (AMETEK Brookfield, Middleborough, MA, USA).

## 3. Results and Discussion

### 3.1. Morphological Study

**Figure 2:**
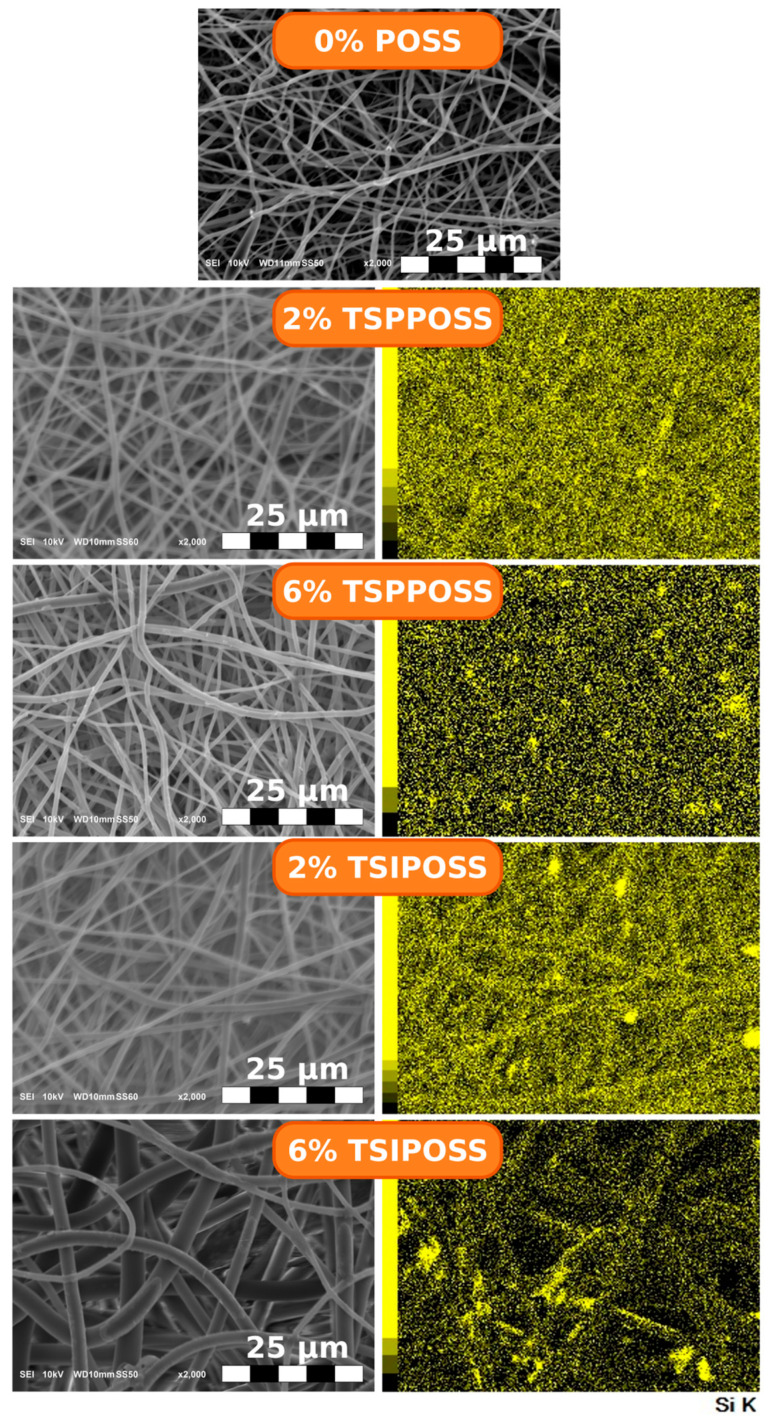
SEM microphotographs of the composite meshes, along with mapping on the K-line of silicon, reflecting distribution of POSS moieties.

The type and concentration of POSS in the neat composite plays an important role in fiber formation (Figure 2). Fibers formed in the electrospinning process of the matrix are mostly characterized by 300–500 nm diameters and arranged in a disordered manner. Addition of 2 wt% of the moiety with aromatic vertex groups (TSPPOSS) leads to the formation of slightly thicker fibers (400–800 nm) with random orientation. However, for the trisilanol POSS with aliphatic vertex group (TSIPOSS), the situation is different. The electrospinning of samples with a POSS concentration of 2 wt% leads to the formation of fibers with a diameter much bigger than that of the neat material. When higher loads of both TSPPOSS and TSIPOSS are present in the electrospun mixture, thicker fibers are formed. The thickest fibers (1–2 µm) can be observed for 6 wt% TSIPOSS-containing material. The mapping on the K line of silicon (Figure 2) shows a good distribution of POSS particles throughout the fibrous matrix. However, several Si-rich regions were found, especially for 6 wt% TSIPOSS. For further investigation of POSS agglomeration, X-ray studies were performed (Section 3.2).

Generally, the addition of POSS nanoparticles to the electrospun system is known to cause a decrease in fiber diameter [36,57]. Interestingly, it has been reported that the presence of silica atoms may increase the conductivity of the solution jet, leading to the formation of finer structures [58]. Moreover, the addition of POSS nanoparticles to the electrospinning mixture can decrease the viscosity, which also influences the process of fiber formation towards thinner structures. However, the majority of studies describe the impact of silsesquioxane nanoparticles added to the polymeric solution as a physical modifier that is not covalently bonded to the polymeric backbone [50,59,60]. Therefore, nanoparticles can easily migrate in the electrospun mixture, resulting in higher conductivity. The current work, however, concerns a system where POSS is covalently bonded to the polymer and thus cannot migrate. Moreover, POSS nanomolecules here cause partial crosslinking, which can hinder both charge and mass flow [61]. This partial crosslinking can also have an impact on the formation of the fibers themselves, making it harder to spin when some of the polymeric chains are interconnected.

**Figure 3:**
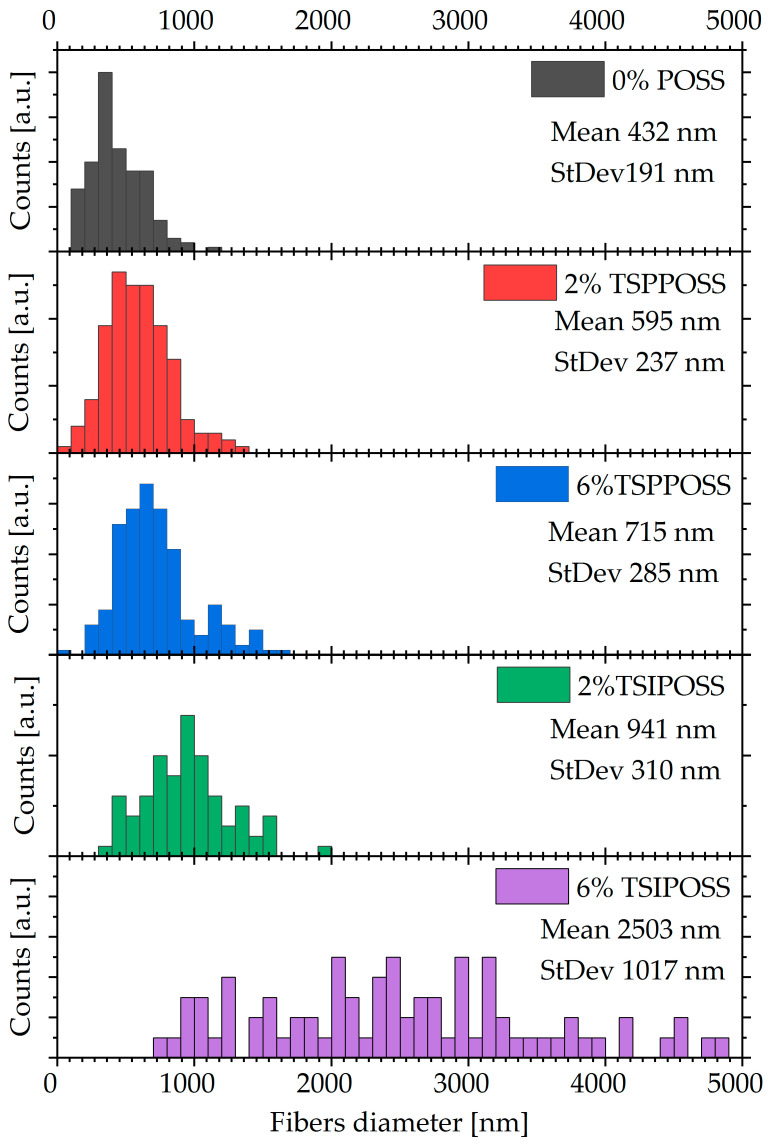
Histograms of fiber diameter distribution.

To quantify the observations described in the previous paragraph, the distribution of fiber diameters was evaluated and shown in the form of histograms in Figure 3.

The above histograms present the fiber diameter distribution in each of the composite meshes. We can observe that the addition of the phenyl-substituted TSPPOSS does not significantly influence the dispersity in the diameter of the obtained fibers. In contrast to that, electrospinning the composite with isobutyl-substituted POSS leads to disturbance in homogeneity, particularly in the case of 6 wt% inclusion. The difference between both silsesquioxane molecules lies in the surrounding cage, which may lead to the conclusion that particles bearing aromatic rings are favorable in electrospinning processes.

### 3.2. X-ray Studies

**Figure 4:**
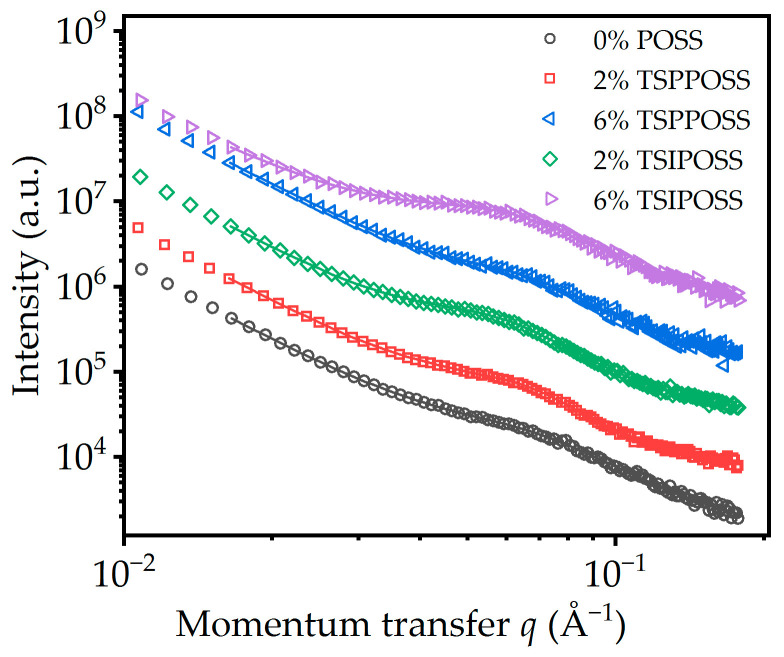
SAXS curves recorded with all materials under investigation. Curves are translated for clarity.

The scattering curve of the matrix shows a weak and broad peak of around 0.08 Å superimposed on an upturn on the low q side (Figure 4). The peak can be attributed to the microphase separation of the liquid crystalline polyurethane inside the fibers, whereas the power law background can be attributed to Porod scattering from larger objects, presumably the fibers themselves. The introduction of POSS in the matrix does not change the general morphology of the curves, which means that there are presumably no new POSS-specific structures in the length scale. However, there is an obvious increase in the intensity of the peak for TSIPOSS composites compared to the matrix. This probably indicates an enhancement of microphase separation.

In lieu of a reliable model for the study of such systems, which tend to show high dispersity in sizes of inhomogeneities, as well as a lack of well-defined shape of the scattering centers, we used the so-called broad peak function to quantify the curves at hand [62]:

(2) $$I_{broadpeak}=\frac{C}{1+{\left(\left|q-q_{0}\right|\xi \right)}^{m}}$$

In this model, $q_{0}$ is related roughly to the distance $d_{0}$ (long period) between inhomogeneities. $\;d_{0}=2\pi /q_{0}$, ξ is the so-called screening length (roughly correlated to the size of inhomogeneities), m is a shape exponent that is associated with the fractal dimension of the inhomogeneities, and C is a strength parameter that increases with contrast and concentration of inhomogeneities.

A Porod term was used for the low q region.

(3) IPorod=Aqn

This term is related to scattering from larger objects. A is a strength parameter and n is an exponent related to the roughness of the surface of these objects [63].

The fit is excellent for all the materials under investigation (Figure 4). The calculation of parameters of the Porod term and the parameters of the broad peak are reported in Table 1.

The Porod model describes scattering from larger objects. Here, it is most likely the fiber itself. Essentially, what we see is the surface of the structure. For smooth surfaces n=4, and smaller values of n denote rough surfaces, i.e., fractal ones. Here n~3, which denotes that the surface of the fibers is very rough [63]. TSIPOSS do not seem to change the characteristic size of the inhomogeneities as quantified by the correlation length ξ, but their distance as quantified by the long period *d*_0_ increases. In addition, modification via isobutyl-substituted POSS (TSI POSS) increases the exponent m. This is a result that is not easy to interpret. Ballard et al. [64] associate an elevated Lorentzian exponent with more tightly packed inhomogeneities. On the contrary, modification by the phenyl-substituted TSPPOSS seems to increase to some extent the size and decrease the distance between inhomogeneities, indicating that this additive significantly promotes phase separation of the materials. This should be associated with the formation of thicker fibers by these composites as observed via SEM. Small-angle X-ray scattering (SAXS) was used for the study of phase separation inside the fibers and the possible presence of nanometric POSS-rich structures.

**Figure 5:**
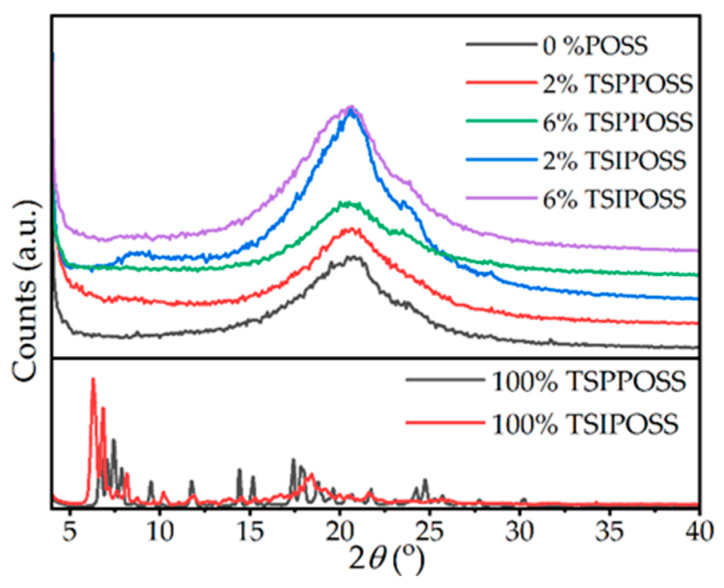
XRD patterns of the composite fibrous mats.

To investigate the crystalline structure and formation of possible POSS-rich regions, XRD studies were performed. The diffractograms of the samples and neat silsesquioxanes are presented in Figure 5.

The pattern of the matrix is mainly amorphous, with the main halo at 22°. Around 2*θ* of 25°, a sharper peak can be observed, which corresponds to the crystalline structure form of a mesogenic unit, which was discussed in our previous works [52,53]. The influence of POSS on this pattern is minimal. No sharp peaks emerge in the patterns of the composite fibers, indicating that the composites are rather amorphous. Broad halos occur at ca. 8° in 2 wt% samples for both POSSs and 6 wt% for TSIPOSS. Their origin can be associated with the crystalline structure of POSS itself, which has main crystalline Bragg peaks around 6°. The abovementioned observations are in accordance with SEM mapping showing Si-rich regions (Section 3.1).

### 3.3. Calorimetric Study

**Figure 6:**
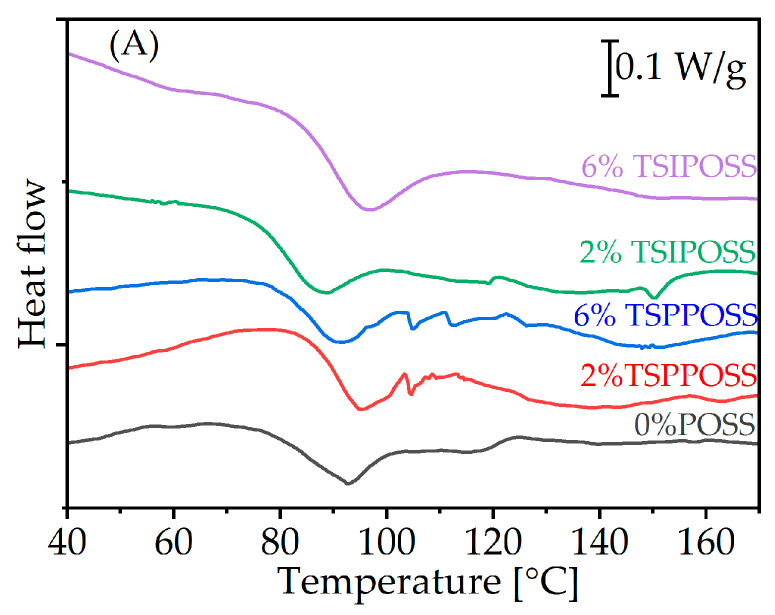

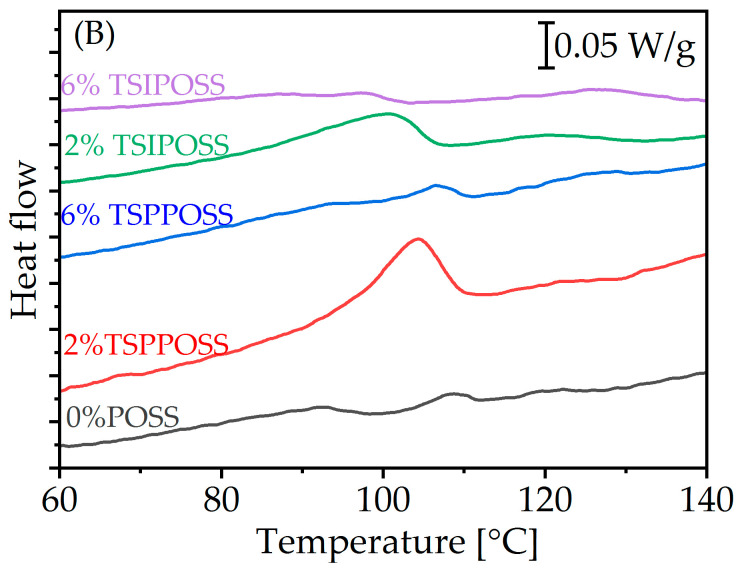
DSC curves recorded in the first heating run (**A**) and subsequent cooling (**B**). Exo up.

The formation of liquid crystalline phases was investigated via differential scanning calorimetry. The DSC curves are presented in Figure 6.

The calorimetric study shows that the obtained fibrous composites have a complex behavior. In the heating run (Figure 6A), the first endothermic peaks in the range of 80–100 °C can be assigned to the melting of solid crystalline structure inside the fiber. For the neat material, such an effect was detected at higher temperatures (120–140 °C).

It can be hypothesized that when a liquid crystalline elastomer is electrospun, crystalline to liquid crystalline phase transition requires less heat to pass through LC phases, possibly due to smaller domains. At higher temperatures, above 120 °C, weaker and broader melting effects can be observed. Interestingly, for a starting material in the heating mode, only a single melting was observed, which can lead to the conclusion that when an LC elastomer is electrospun, more than one type of crystalline or liquid-crystalline domain is formed. Perhaps fast solvent evaporation during electrospinning causes the formation of smaller domains because they do not have as much time to organize themselves as they do during slow evaporation in the elastomer synthesis process [65].

In the cooling run (Figure 6B), a faint crystallization effect is visible for the matrix and both 6 wt% POSS composites. More intense ordering effects appear for 2 wt% loadings as exothermic peaks. Promoting crystallization is a well-known effect for polymer/POSS systems [34,66]. However, it seems interesting that it occurs only for smaller additions and does not depend on the structure of the silsesquioxane cage. The reason behind this behavior is unclear at this point. It is possible that the higher degree of chemical crosslinking expected for higher POSS loadings hinders the ability of chains to freely orient and organize toward the formation of a stable secondary hydrogen bond network.

Interestingly, there is no correlation of peak temperature with long period as is known for lamellar crystals or in polyurethanes [67]. It is possible that the differences in fiber sizes and corresponding changes in heat transfer have an impact on the temperature of the apparent order to disorder temperature. In any case, the reason for this discrepancy is an interesting point to follow in future works.

### 3.4. Thermomechanical Study

**Figure 7:**
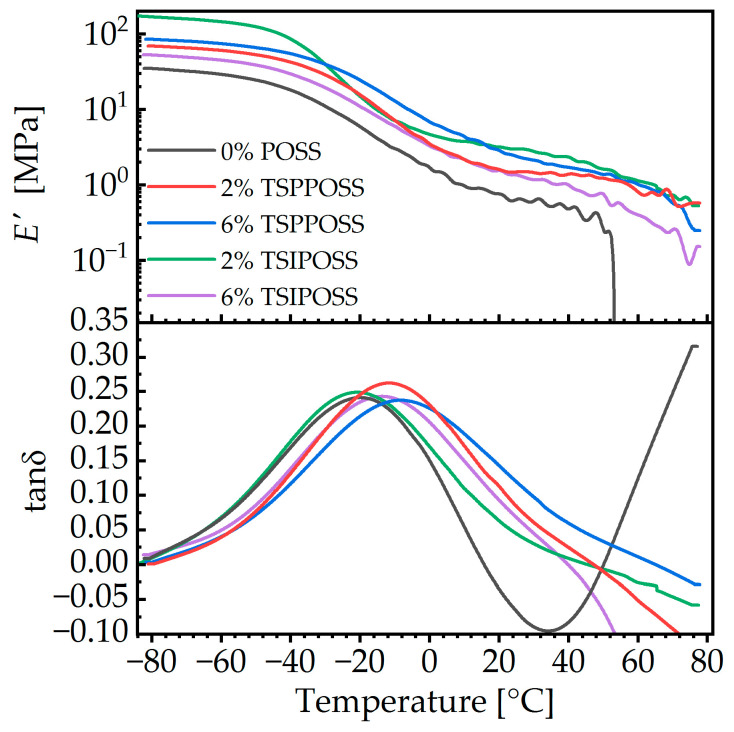
DMA results for the as-prepared nanocomposite meshes.

All composite meshes show a modulus higher than that for neat material (Figure 7). The glass transition temperature $T_{g}$, the maximum value of tanδ, and Young’s modulus in the glassy and rubbery states data are shown in Table 2.

Reinforcement of the modulus after the incorporation of POSS is a well-known phenomenon on the nanoscale in polyurethanes [68,69,70]. The effect can be discussed on several length scales: on the atomic scale, the impact of applied POSS nanoparticles on mechanical properties is mainly due to the partial crosslinking of polymer chains with a three-functional structure. On the nanoscale, the stronger microphase separation in the hybrids can contribute to that effect, along with the incorporation of rigid siliceous cores. On the micrometer scale, the higher thickness of fibers formed by the composites might also have some effect. A difference between the applied silsesquioxane and its load can also be observed. Aromatic-substituted POSSs enhance Young’s modulus when their amount increases. In the case of isobutyl-substituted POSS composites, the highest reinforcement can be observed for 2 wt% content, giving also the best results among all the composites. Such observations prove that the POSS cage structure can play an important role in the improvement of mechanical properties.

The glass transition temperature, as quantified by the peak temperature of tanδ curves, increases by several degrees with increasing POSS content. The effect is more pronounced for the phenyl-substituted moiety. Such an increase in the glass transition temperature is associated with a slowing of dynamics. In polyurethane POSS systems, several mechanisms may give rise to this effect: (i) the restriction of dynamics due to crosslinking; (ii) morphological changes, that is, decrease in microphase separation; (iii) restriction of dynamics due to the participation of POSS in the glass transition, in the sense of mixing models, such as Fox; and (iv) anchoring of polymer chains on nanoaggregates [71]. According to the SAXS results (Section 3.2), there are no nanometric POSS aggregates in the system, so (iv) can be eliminated. The degree of crosslinking should be comparable between the two types of POSS, as they possess the same chemical functionality. However, the effect on phase separation is different for the two types of vertex groups. Systems based on isobutyl-substituted moieties seem to promote the formation of more and larger inhomogeneities, whereas those based on phenyl-substituted ones tend to decrease their number. Furthermore, phenyl-substituted POSS moieties (more correctly their homopolymer) are known to have a fairly high glass transition temperature, over 200 °C [72], whereas isobutyl-substituted ones are predicted to have a *T_g_* in the vicinity of 0 °C [71]. This observation is compatible with the more intense increase in *T_g_* upon the addition of phenyl-substituted POSS, if we assume that the dominant mechanism is the third one, i.e., the participation of diluted POSS in the glass transition.

### 3.5. Mechanical Study

**Figure 8:**
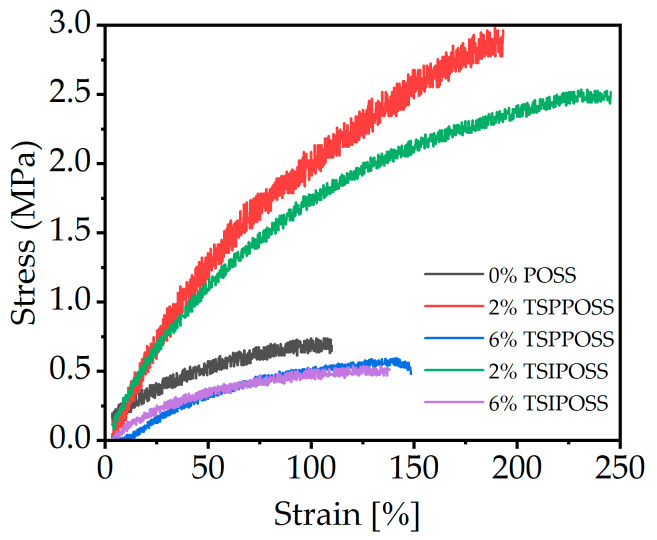
Tensile strength plot of LCPU/POSS nanocomposite meshes.

The measurement of tensile strength shows the reinforcement effect of both types of POSS on the LCPU matrix (Figure 8).

We found that 2 wt% POSS loading in composites leads to a significant increase in tensile strength at break by more than 500%, along with increased elongation at break. Interestingly, when 6 wt% of POSS materials are electrospun, the resulting meshes exhibit much lower tensile strength—lower than that for neat samples. This should be correlated with the higher degree of ordering in the 2 wt% materials, as observed via DSC. Moreover, the mesoscopic morphology of the fibrous mat may play a role [73]. It is hypothesized that two different mechanisms may play a role in the reinforcement of the LCPU fibers by POSS. First, for 2 wt% silsesquioxane loading in composite meshes, the average diameter of the fibers does not differ much from that of the neat material, reaching a few hundreds of nanometers. For this size of fiber, reinforcement on the molecular scale via partial crosslinking by POSS may play a crucial role. When the size of the fibers increases in the case of electrospinning of 6 wt% loadings of POSS, the small-scale reinforcement is surpassed by a macroscale effect of the fibers themselves. Thicker fibers with fewer entanglements exhibit a weaker mechanical response, as they are easier to break. Another possible explanation can be found on calorimetric curves. Hybrids of 2 wt% exhibit better phase separation, which has a significant impact on polymer reinforcement [74]. Compared to the data for starting material in bulk reported in a previous work [53], some changes can be observed. For TSPPOSS, the highest reinforcement was recorded for the 6 wt% load of additive and for TSIPOSS the 4 wt% (not discussed in this paper). In both series of composites, the mechanical response of 2 wt% material did not differ much from that of the neat material. It can be thus assumed that POSS moieties affect mechanical properties rather indirectly through their influence on micro- and macromorphology.

## 4. Conclusions

Fibrous mats were prepared via electrospinning hybrid organic–inorganic liquid crystalline polyurethanes (LCPUs) modified using POSS as crosslinking agents. It was possible to obtain fibers with diameters in the sub-micrometer range. The spatial distribution of POSS moieties within the fibers was high, despite the occurrence of rare aggregates with sizes in the order of micrometers. Crosslinking with POSS resulted in somewhat thicker and more disperse fibers. The type of organic vertex groups of POSS moieties played an important role in this. That is, the isobutyl-substituted moieties had only a small effect, while the phenyl-substituted POSS caused a much more pronounced one, resulting in a content of 6 wt% in a very broad distribution of diameters in the order of μm.

This should be correlated with the internal micromorphology of the fibers. Although SAXS detected no specific POSS-related inhomogeneities at the nanoscale, the microphase separation changed significantly. Isobutyl-substituted POSS caused larger and less distanced mesogen-related inhomogeneities than the matrix, and phenyl-substituted ones resulted in inhomogeneities of similar size as those of the matrix but significantly more distanced from each other. The surface of the fibers was found to be rather rough, but POSS had no effect on that. On the atomic scale, there was only very minor indication of POSS crystallinity, despite the presence of some weak and broad peaks, possibly related to the micrometric aggregates observed via scanning electron microscopy.

The glass transition temperature increased with crosslinking, with the benzene-substituted POSS having a more pronounced effect. This is due to the combined effect of deceleration of dynamics due to crosslinking, which is presumably the same in both types of composites, and changes in microphase separation, which are more pronounced in the case of the phenyl-substituted POSS. The slower mobility of the POSSs themselves possibly plays a role too.

The mechanical modulus and elongation at break also increased with POSS incorporation, but interestingly, the effect is more pronounced when a smaller amount of POSS is introduced into the system.

## Figures and Tables

**Table 1:** Parameters of the Porod and broad peak terms. Details in text.

Sample	*m*	$q_{0}$ [Å^−1^]	ξ [Å]	$d_{0}$ [Å]	*n*
Matrix	2.47 ± 0.19	0.0458 ± 0.0027	26.2 ± 1.6	137 ± 8	3.185 ± 0.029
2% TSP	2.68 ± 0.09	0.0516 ± 0.0004	37.7 ± 0.7	122 ± 1	3.066 ± 0.010
6% TSP	2.29 ± 0.19	0.0542 ± 0.0009	35.4 ± 1.7	116 ± 2	2.982 ± 0.013
2% TSI	3.54 ± 0.42	0.0341 ± 0.0049	26.6 ± 3.2	184 ± 27	3.289 ± 0.014
6% TSI	3.25 ± 0.57	0.0340 ± 0.0080	23.1 ± 3.9	184 ± 44	2.950 ± 0.047

**Table 2:** Glass transition temperature $T_{g}$, maximum value of tanδ, and Young’s modulus in the glassy and rubbery states, as determined via DMA.

Sample	$tan\delta_{max}$	$T_{g}$ [°C]	*E’* [MPa] (at −60 °C) Glassy State	*E’* [MPa] (at 25 °C) Rubbery State
Matrix	0.24	−20	29.3	0.6
2% TSP	0.26	−12	61.9	1.5
6% TSP	0.23	−9	74.8	2.4
2% TSI	0.25	−21	146.0	3.0
6% TSI	0.24	−11	45.3	1.3

## Data Availability

Data are available on request.
